# A Comparison of Experimental Methods to Induce Mental Fatigue

**DOI:** 10.1177/00315125261420694

**Published:** 2026-02-11

**Authors:** Margit M. Bach, Kerstin Brandstetter, John Stins, Peter J. Beek

**Affiliations:** 1 Department of Human Movement Sciences, Faculty of Behavioural and Movement Sciences, Institute of Brain and Behavior Amsterdam and Amsterdam Movement Sciences, 1190 Vrije Universiteit Amsterdam, Amsterdam, The Netherlands; 2 Center for Neuroplasticity and Pain (CNAP), Department of Health Science and Technology, The Faculty of Medicine, Aalborg University, Gistrup, Denmark

**Keywords:** mental fatigue, dual task, tloaddback, cognitive fatigue, N-back, validation

## Abstract

Appropriate induction of mental fatigue in the lab is imperative for properly investigating the effects of mental fatigue on behavior. In this study, we aimed to (1) identify a task for inducing mental fatigue that is both effective (i.e. sufficiently demanding) and feasible (i.e., sufficiently short in duration), and (2) to assess its effects on boredom, loss of motivation, and sleepiness. Therefore, we compared the following tasks: (1) an individualized TloadDback task (16 minutes), (2) an individualized TloadDback task (32 minutes), (3) an N-back task (32 minutes), and (4) a control task in the form of watching a documentary (32 minutes), using a within-participants design with 30 participants. Subjective and objective outcome measures were used to evaluate levels of fatigue, boredom, motivation and sleepliness pre- and post-task. While all investigated tasks induced mental fatigue, the individualized TloadDback struck the best balance between effectiveness and feasibility and should therefore be preferred in experimental research.

## Introduction

Fatigue is considered a major threat to human performance. We here focus on mental fatigue, which is commonly defined as a state characterized by subjective feelings of fatigue, caused by (prolonged) periods of cognitive activity (Brahms et al., 2022; Jacquet et al., 2021). Mental fatigue was defined in this study as a feeling of reduced focus, concentration, and presence of mental fogginess, and is different from sleepiness. Mental fatigue may hamper productivity and even have serious consequences in some occupations. Think about the flight leader who must keep focus on the task at hand to avoid any potential crashes, or the surgeon performing a risky and challenging operation. Mental fatigue can arise not only from prolonged cognitive activity and alertness, as one might expect, but also from brief tasks involving high mental workloads with low rewards (Boksem & Tops, 2008). Mental fatigue becomes manifest as the feeling of tiredness and exhaustion, accompanied by a lack of concentration and performance loss (Boksem & Tops, 2008; O’Keeffe et al., 2020).

Mental fatigue is also known as cognitive fatigue (Mangin & Pageaux, 2024; Pageaux & Lepers, 2018), and some authors make a further distinction between “active” and “passive” fatigue (Mangin & Pageaux, 2024; Pickering et al., 2024), where “active” fatigue stems from a task requiring continuous attention during task execution and “passive” fatigue results from prolonged sustained attention and understimulation. Here the term mental fatigue is used.

Expedient induction of mental fatigue in experimental studies is imperious to being able to consistently measure markers of mental fatigue and investigate its effects on task performance. Several attempts have been made to find an optimal task to induce mental fatigue (Borragan et al., 2017; Hassan et al., 2024; O'Keeffe et al., 2020; Smith et al., 2019). To improve reliability and feasibility, the task used to induce mental fatigue should be (1) effective in inducing mental fatigue (subjective feelings of mental fatigue as well as objective reductions in cognitive performance), and (2) practically feasible, e.g., not take too much time to prepare and administer in a research setting. There have been several attempts in pertinent literature to determine which factors co-vary with changes in mental fatigue. It has often been observed that mental fatigue is accompanied by sleepiness, boredom, loss of motivation, changes in mood, etc. (Brahms et al., 2022). However, it is unclear whether these variables are causal constituents of mental fatigue or not. For example, Borrogan et al. (2017) argued that cognitive fatigue is independent of sleepiness. Although this makes sense from a theoretical perspective, in practice many of these variables (which are often measured subjectively) tend to co-vary. Mangin and Pageaux (2024) asserted that, independent of one’s theoretical stance, it is important to monitor variables such as boredom and sleepiness when investigating mental fatigue. We agree with this assertion and consider it pertinent to know how these factors are affected by the task used or selected for inducing mental fatigue. We therefore added an exploratory analysis to investigate how boredom (i.e., a state of underarousal), loss of motivation, and sleepiness (i.e., an increase in sleep pressure) are affected by the task at hand.

Several tasks have been used to experimentally induce mental fatigue. Some studies have also directly compared different tasks to examine how objective and subjective markers of fatigue are affected. The most common tasks to induce mental fatigue in experimental studies have been various types of N-back tasks (Chen et al., 2015; Lin et al., 2014; Magnuson et al., 2021; Shigihara et al., 2013), the AX-CPT task (Hachard et al., 2020; Noe et al., 2021; O'Keeffe et al., 2020; Smith et al., 2019), the Stroop task (Martin et al., 2019; Rubio-Morales et al., 2022; Smith et al., 2019), and the Time Load Dual-back task (TloadDback task; Borragan et al., 2017; Borragan et al., 2016; Jacquet et al., 2020; O'Keeffe et al., 2020; O'Keeffe et al., 2021). The N-back task is a singular task and can be comprised of both numbers and letters. The task for the subject is to determine whether the current stimuli matches a set of previous (N = 1/2/3) stimuli by pressing a button. The AX-CPT task is another singular task, where the objective is to identify when the letter X follows a letter A in a series of letter sequences. The Stroop task is a (verbal or visual) discrepancy task in which there is a discrepancy between the written word and the color of the word. Both the Stroop task and the AX-CPT not only involve sustained vigilance, but also response inhibition. The TloadDback task is, contrary to the N-back task, AX-CPT, and Stroop tasks, a dual-task, consisting of a combination of a parity task and a 1-back task, and administered by showing an alternating series of single letters (A, C, E, L, N, P, R, T, U) and digits (1, 2, 3, 4, 6, 7, 8, 9) on the screen. It has the advantage that it can be adjusted to each individual participant (Borragan et al., 2016, 2017) and kept at short duration ( ∼ 16 min). The main advantage of the TloadDback task is the individualized speed of stimulus presentation, and hence task complexity. Before administering the task, a test is done to find the shortest stimulus time duration (STD) where an accuracy higher than 85% is still maintained. This STD is expected to differ between individuals for various reasons. Interestingly, O’Keeffe et al. (2020) found that both the AX-CPT (administered for 90 minutes) and TloadDback (16 minutes) reliably induced mental fatigue, but that the AX-CPT (and not TloadDback) additionally reduced physiological arousal (suggestive of boredom and sleepiness). This dissociation led the authors to conclude that the TloadDback is better suited to study the effects of mental fatigue in isolation.

For the task to successfully induce mental fatigue, it is imperative that its duration remains manageable, thereby allowing for straightforward application in an experimental research setting. The duration of the mentally fatiguing task is arguably equally important as the type and complexity of the task provided. Hassan et al. (2024) recently developed a comprehensive two-hour test battery combining various simple and complex tasks, which led to increased subjective feelings of mental fatigue and a decline in performance. Nevertheless, the long duration of the test battery renders its application cumbersome and less practical. In contrast, the short, individualized TloadDback task (16 minutes) has shown to be useful in inducing subjective feelings of mental fatigue (Borragan et al., 2017; Borragan et al., 2016; O’Keeffe et al., 2020; Weissinger et al., 2024). However, Jacquet et al. (2020) questioned whether 16 minutes was long enough to induce subjective feelings of mental fatigue as well as objective signs thereof, such as decreases in performance. O’Keeffe et al. (2020) found a difference between the short task and the long task, suggesting that task duration influences the degree of mental fatigue. However, their comparison between a highly complex task of 16 minutes and a long, simple task of 90 minutes was not ideal, as two dimensions, namely task complexity and duration, were manipulated together, making it impossible to determine the critical factor for any observed difference. Two tasks with similar durations, one simple and one complex, would allow for assessing the effect of both factors. Because the N-back task is so widely used in the mental fatigue literature, and because it has several features in common with the TloadDback task, a direct and methodologically sound comparison between the two is indicated. In this study we fill this research gap by including two tasks that are equal in duration but different in task complexity.

In many studies, mental fatigue is assessed using both subjective and objective outcomes. Subjective feelings of mental fatigue are best assessed with a VAS (Smith et al., 2019). VAS has been used extensively as a method for assessing the presence of mental fatigue (e.g., in Borragan et al., 2017; Borragan et al., 2016; O’Keeffe et al., 2020; O'Keeffe et al., 2021; Smith et al., 2019; Weissinger et al., 2024). More objective measures are motivated by the alleged depletion of cognitive resources with time on task and/or cognitive effort invested in task performance. To this end, reaction time and/or reaction time variability (assessed directly after the fatiguing task) are considered valid markers (Behrens et al., 2023; Boksem et al., 2005; Smith et al., 2019).

In sum, we aimed to find a mentally fatiguing task with a balance between effectiveness (i.e. complexity) and feasibility (i.e. duration), and to assess effects on boredom, reduction in motivation, and sleepiness. We therefore compared an individualized 16-min TLoadDback task, an individualized 32-min TloadDback task, a 32-min N-back task, and a self-chosen documentary as control task, while monitoring their effects on boredom, lack of motivation, and sleepiness. We hypothesized that a short, individualized task for inducing mental fatigue would be more effective and suitable than a simpler or longer task.

## Methods

### Protocol

A within-subject design was used, whereby each participant presented to the laboratory on five separate occasions, i.e., days: one session to familiarize themselves with the tasks and questionnaires, one control session, and three experimental sessions during which the participants were mentally fatigued. The order of the mentally fatiguing sessions as well as the control tasks was randomized over participants to minimize the risk of possible order effects, using a random number generator in MATLAB (The Mathworks, Natick, MA, United States, ver. 2020a).

The only inclusion criterium was that participants needed to be healthy and older than 18 years; no exclusion criteria were used. All participants were asked to fill out an online version of the Morningness-Eveningness Questionnaire (Horne & Ostberg, 1976) prior to scheduling the first session. The Morningness-Eveningness questionnaire comprises 19 questions assessing the circadian rhythm of the participants; the scores on this questionnaire determine whether a participant is a so-called morning-person, evening-person or in-between. The results (moderate morning, intermediate, or moderate evening) were then used to schedule lab sessions at each participant’s most optimal time of day, with sessions preferably spaced within a few hours of each other on separate days (time between sessions ranged from 1 to 30 days, 4.4 days on average with a standard deviation of 4.5 days). Participants scoring “moderate morning” (n = 4) were scheduled between 09:00 and 12:00 with the median session starting at 09:55; “intermediate” participants (n = 22) were scheduled between 09:00 and 16:00 with the median session starting at 12:00, and finally the participants scoring “moderate evening” (n = 4) were scheduled between 11:00 and 17:00 and the median session starting at 14:45, with one exception in which a session started at 08:30 as this was the only opportunity for the participant in question to finalize the study.

Participants were asked to refrain from ingesting any caffeine within two hours of each session which was checked before the start of each session.

The three mentally fatiguing tasks were two versions of the TloadDback task (Borragan et al., 2016, 2017), and an N-back task. The TloadDback task is a working memory task, which was originally developed to be administered for 16 minutes (here abbreviated as: TLoad16). To compare whether prolonged duration resulted in increased MF, it was also administered for 32 minutes (Tload32).

The TloadDback task is a dual task consisting of a combination of a parity task and a 1-back task. A series of single letters (A, C, E, L, N, P, R, T, U) and digits (1, 2, 3, 4, 6, 7, 8, 9) alternate on the screen. For the letters, the task is to determine whether the letter is the same as the previous letter (1-back) and for the digits, the task is to determine whether it is even or odd. The responses of the participants were registered with key presses.

Additionally, the N-Back (N = (2) task with letters (A, C, E, L, N, P, R, T, U) for 32 minutes (N-Back), and finally, the control task consisted of watching 32 minutes of the beginning of 1 of 3 documentaries of own choice (CTRL). See Figure 1 for an overview of the protocol.

**Figure 1.**
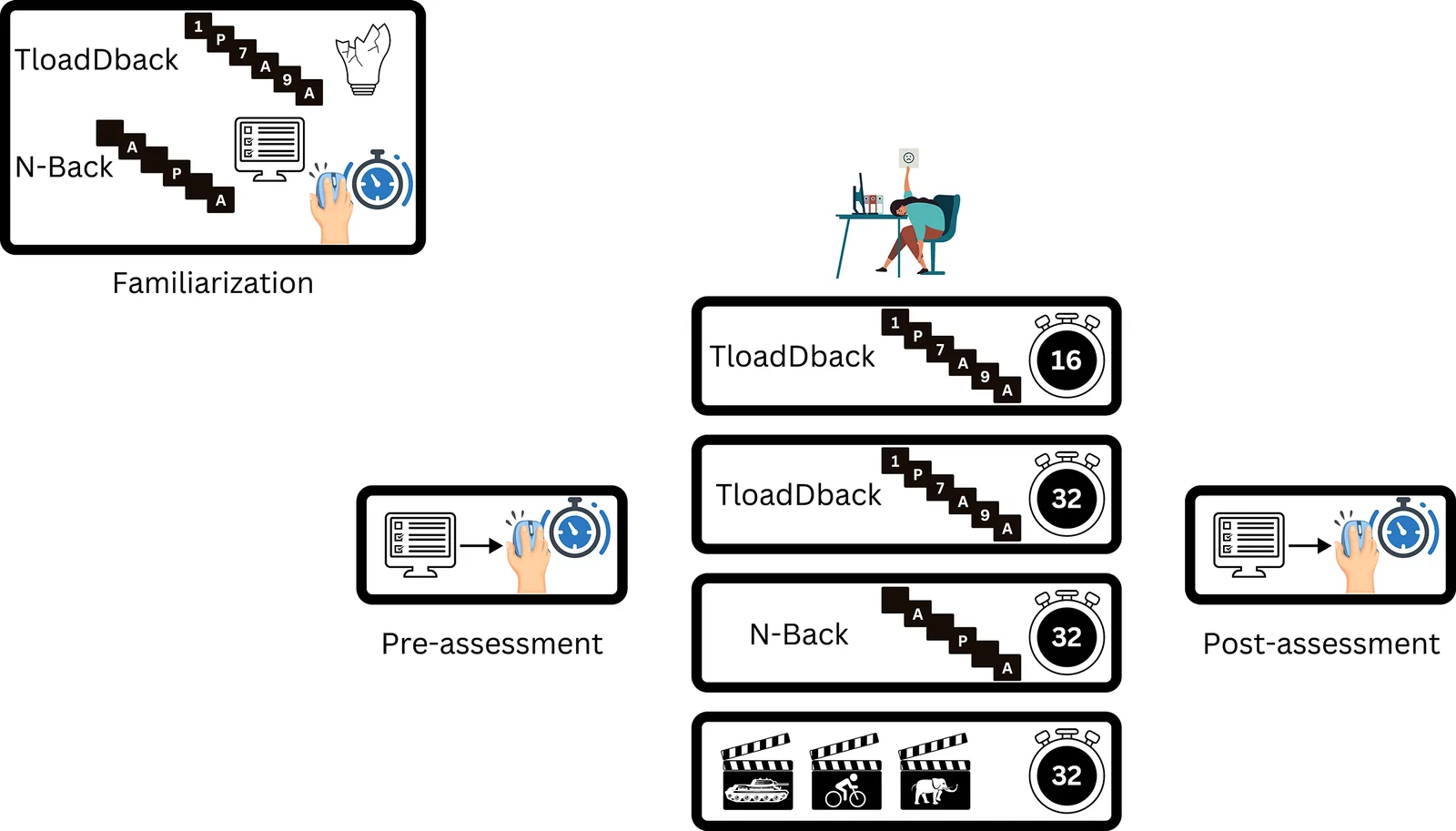
Protocol: All participants conducted a familiarization session during which their individual level of the TloadDback task was determined, and they were introduced to the subjective measures, reaction time task, and the N-Back task. In the following sessions, there was a pre-assessment consisting of a questionnaire and a reaction time task, followed by one of the mentally fatiguing tasks or the control tasks, and finally ended with a post-assessment with subjective measures and reaction time task. The order of tasks was randomized over participants to ensure a balanced design

### Participants

A sample size calculation was performed using the ANOVA_power tool (Lakens & Caldwell, 2021). A simulation was run using a 4-within design, consisting of the three mentally fatiguing tasks and one control task. The means for the four factors were based on known research as of October 2022 (when the study was planned) and based on the pre-post change of the visual analog scale (VAS) of mental fatigue. Means of 2.5, 1.8, 1.2, and 1.0 cm were used as a proxy for the expected VAS scores. A mean of 2.5 cm for the TLoad16 was based on Borragan et al. (2017), 1.8 cm for NBack (Ishii et al., 2013), and 1.0 cm for the control task (O'Keeffe et al., 2020) were used. To account for the lack of known research for a 32-min version of the TLoadDback task in October 2022 when the power analysis was performed, the 16-min low cognitive load condition from Borragan et al. (2017) was used as a proxy. In the design of the power analysis simulation, a standard deviation needs to be specified. To this end, a common standard deviation of 1.03 (default value) for all correlations among within-subject factors of 0.5 (default value) was used, as well as a standard deviation of 1.8 (based on the same studies). A simulation (2000 iterations) with no sphericity correction (default value) and an alpha-level of 0.05 was run. As the ANOVA was deemed exploratory, a Bonferroni correction was chosen for multiple comparisons for the post-hoc t-test comparisons of the four factors. The simulations revealed that with a sample size of 16 we would have a power of nearly 100% with an effect size (partial-eta squared) of 0.48 for the full model (or power of 85%, and an effect size of 0.25 with the higher standard deviation). Since we were not only interested in the to-be-expected effect on subjective mental fatigue, but also in other outcome measures, we decided to nearly double the sample size to 30.

Participants were recruited via a student credit system as well as online posters and word-of-mouth. Some participants received student credits for their participation while others received a participation fee of 10€ per hour. All participants signed an informed consent form prior to their participation.

To manage levels of motivation, participants were informed that performing in the top 10% (without providing further elaboration on what this entailed) would lead to a 10€ reward as either an addition to the participation fee if they received one or as a stand alone payment. As all participants would receive this reward upon completion of the study, no efforts were made to calculate this performance measure as it was used solely as an attempt to keep participants engaged in the tasks.

## Familiarization Session

Each participant conducted a familiarization session that consisted of signing the informed consent forms, familiarizing themselves with the subjective measures, answering questions about handedness, age, and gender, finding their individual level of the TloadDback task, and familiarizing themselves with the N-Back task and the reaction time task.

First, the participants were familiarized with the subjective measures. Subsequently, the participants familiarized themselves with the TloadDback task, which was done in several stages as in previous studies (Borragan et al., 2017; Borragan et al., 2016; O'Keeffe et al., 2020). The TloadDback task is a cognitive dual task combining a parity task with a 1-back task. The different components of the task were practiced separately before being combined and finally repeated at increasing speed until the participant could no longer maintain >85% accuracy. The parity task involved determining whether a digit was even or odd, using the right hand. When an even digit appeared on the screen, the number 2 on the numpad had to be pressed and in case of an odd digit, the number 3 on the numpad had to be pressed. Next, the letters were practiced. In this 1-back task, the participants were instructed to decide if the current letter was the same as the previous letter, and when it was, to press the space bar with their left hand. Finally, the two tasks were combined, i.e., letters (A, C, E, L, N, P, R, T, U) and digits (1, 2, 3, 4, 6, 7, 8, 9) alternated on the screen. After completion of the practice rounds, the participants began the individualization part of the task in which every 60 stimuli (30 letters and 30 digits), the stimulus duration time (SDT) either increased or decreased in steps of 100 ms (depending on their performance). The starting stimulus was 1.2 seconds, the number of blocks ranged between 3 and 14 with a median of 10. The participants were encouraged to take breaks between each block; they could decide themselves how long these breaks were. After familiarization with the TloadDback task, participants were familiarized with the reaction time task (3 minutes) and finally with the N-Back task (3 minutes).

### Experimental Tasks

The experimental tasks were programmed in MATLAB (The Mathworks, Natick, MA, United States, ver. 2020a). Both the TloadDback task and the N-Back tasks were based on the code downloaded from https://osf.io/ay6er/ and Psychtoolbox (Brainard, 1997; Kleiner et al., 2007), but was improved in terms of speed, accuracy, and adjusted to fit our own experimental setup. White letters/digits were presented on a black screen with a refresh rate of 240 Hz located 55 cm from the participant. A mechanical keyboard (CORSAIR K55 RGB Pro Keyboard) with a transfer rate of 1 kHz was used to register keypresses.

In the TloadDback tasks, the SDT was based on the SDT as determined during the familiarization session. For the N-Back task, the stimulus (letters used were: A, C, E, L, N, P, R, T, U) was presented for 500 ms with an inter-stimulus interval of 1000 ms (black screen).

Performance (speed and accuracy) were automatically recorded but not presented here, since we deemed them not important for the purpose of this study.

Watching a documentary has been used as a control task in several studies (Hachard et al., 2020; Martin et al., 2019; O'Keeffe et al., 2020; Smith et al., 2019; Weissinger et al., 2024). However, a disadvantage of using a documentary, as highlighted by O’Keeffe et al. (2020), is that it may lead to underarousal and boredom. Concerningly, O’Keeffe et al. (2020) reported that 60% of participants were bored from watching the documentary. Having participants select their own documentary for watching may decrease the risk of boredom/underarousal during the control task. Participants could choose between 3 different documentaries (“Icarus” (Fogel, 2017); cycling/doping, “Surviving Paradise – A family Tale” (Godfrey & MeechDirector., 2022); nature, and “Greatest Events of WWII in Colour” (Boxer, 2019); history) to present the opportunity for every participant to find a documentary of their interest. For each documentary, the first 32 minutes were shown irrespective of the narrative arc and original duration of the documentary.

### Subjective Measures

At the beginning of each experimental session (prior to the reaction time task) and immediately after completion of each experimental task, the participants had to answer questions that were administered via Qualtrics (Qualtrics XM, London, England). To rule out ingestion of caffeine before sessions, participants were also asked about any (and timing of) intake of caffeine prior to the session as part of the pre-assessment. After having completed the subjective measures, they performed the reaction time task.

## VAS

To assess the pre-levels of mental fatigue, boredom, and motivation, visual analogue scales (VAS) ranging from 0-100 without any number markings were used. To assess mental fatigue, the participant was asked to assess the following: ‘Please rate the level of mental fatigue you experience right now’, where mental fatigue was described in the question as follows: “Mental fatigue is a state of exhaustion and decreased cognitive functioning, leading to reduced focus, concentration, and presence of mental fogginess. It differs from sleepiness, which reflects the body’s need for restorative sleep”. The endpoints were marked with ‘no mental fatigue’ and ‘worst possible mental fatigue’. Boredom was assessed by asking the following question: ‘Please rate the level of boredom you experience right now’ with the endpoints of ‘no boredom’ and ‘worst possible boredom’. Finally, motivation was assessed by asking the following question: ‘Please rate the level of motivation for this study’ with the endpoints of ‘no motivation’ and ‘highest possible motivation’. During the post-assessment, the VAS for mental fatigue, boredom, and motivation, and the SSS were repeated.

### Sleepiness

To assess the current level of fatigue, the Stanford Sleepiness Scale (SSS; Hirshkowitz & Sharafkhaneh, 2017; Hoddes et al., 1973) was used. The SSS is a Likert scale with 7 points ranging from 1 – “feeling active and vital, alert or wide awake” to 7 – “No longer fighting sleep, sleep onset soon; having dream-like thoughts”.

### Rating Scale Mental Effort and NASA-TLX

In addition to these subjective ratings, the Rating Scale Mental Effort (RSME; Zijlstra, 1993, Zijlstra & van Doorn, 1985) and NASA-TLX (Hart & Staveland, 1988) were administered. These two scales are commonly used to assess the levels of mental workload (Hancock et al., 2021; Longo et al., 2022). RSME is a scale ranging from 0-150 mm asking the participants to ‘please rate the extent of mental effort you needed to perform the computer task by clicking on the scale’ with qualifiers ranging from “absolutely no effort” (0) to “extreme effort” (∼112) and is brief to administer. NASA-TLX was used as it is widely employed in previous studies investigating mental workload (Longo et al., 2022). The NASA-TLX was used in its simplest form, and thus a score for each of the six domains (Mental demand, Physical demand, Temporal demand, Performance, Effort, and Frustration) was calculated separately (Bolton et al., 2023). The performance score was the opposite of all other scores (low score equals good performance), but this was reversed for analysis.

### Objective Measures

To assess the reaction time pre- and post-experimental task, a 3-min psychomotor vigilance task (PVT; Basner et al., 2011) was used. PVT is a reaction time test in which participants react as fast as possible to a stimulus presented at the screen with random inter-stimulus intervals (ISI) ranging from 2-10 seconds. Reactions faster than 100 ms were counted as “false starts” and reactions slower than 1s were counted as “missed” and excluded from further analysis. In total 10 trials across all sessions and participants were slower than 1s and removed from analysis. Each participant performed an average of 28.6 trials per session and the total number of trials missed in any one session was 10% of the trials, totalling 3 trials. The average reaction time (PVTm) and the standard deviation of the reaction time (PVTstd) were analysed for each participant. The PC-PVT 2.0 (Khitrov et al., 2014) was used to administer the task and in combination with a gaming mouse (CORSAIR Sabre RGB Pro Gaming) with a transfer rate of 8 kHz.

## Statistics

A repeated measures ANOVA (rmANOVA) was used to assess the influence of subjective and objective measures on the four different tasks. For the subjective measures mental fatigue, boredom, motivation, and sleepiness and the objective measures PVTm and PVTstd, the change scores ( change=post−pre ) were used as the factor in the rmANOVA. Change scores were used to assess the change across time – it was determined post hoc that no differences existed between tasks at baseline. Effect sizes are reported at partial-eta squared ( $\eta^{2}$ ). For the subjective measures RSME and each of the six domains of the NASA-TLX the factors were the post scores. When a significant result was found, a Bonferroni post hoc test was used to assess differences between conditions. Normality was determined by a non-significant Shapiro-Wilk test and complimented by a visual inspection of the Q-Q plots. In case of non-parametric data, a Friedman test was used and if a significant main effect was present, the Durbin-Conover’s post hoc test.

To assess whether there was a significant difference between the pre- and post-scores for each condition for the subjective and objective measures, a paired t-test was used and a p-value of 0.01 was considered significant to correct for multiple comparisons ( α= 0.05/4). Statistics were performed in JASP (JASP Team, 2023) and MATLAB.

Means and standard deviations are presented unless stated otherwise.

## Results

Thirty-one participants were recruited to participate in the study with one participant dropping out after the familiarization session. No reason was provided. Of the 30 remaining participants, one participant could not attend the control session; the data for this participant collected during the other sessions was included in the analysis. Of the included participants, 20 identified as female, 9 as male and one as non-binary. They were aged 28.1 ± 9 years (mean ± std), and 26 were right-handed. No one reported ingesting any caffeine within two hours of any session.

The three different documentaries were chosen at an almost even split, with the nature and cycling/doping both chosen ten times and the history documentary chosen nine times.

The SDT of the TloadDback task ranged between 0.6 and 1.5 s with a median of 0.8.

### Subjective Measures

There was a significant effect of task ( $F\;\left(3,84\right)=15.49,p<0.0001,\eta^{2}=0.23$ ) on the subjective levels of mental fatigue, see Figure 2. Post hoc testing using the Bonferroni correction revealed that the change scores of all three experimental tasks were all significantly higher compared to the control task (M = 19.11 ( p=0.002 ), 33.57 ( p < 0.0001 ), 24.77 ( p < 0.000 1) for TLoad16, TLoad32, and the N-Back tasks, respectively), in the absence of significant differences between the three experimental tasks.

**Figure 2.**
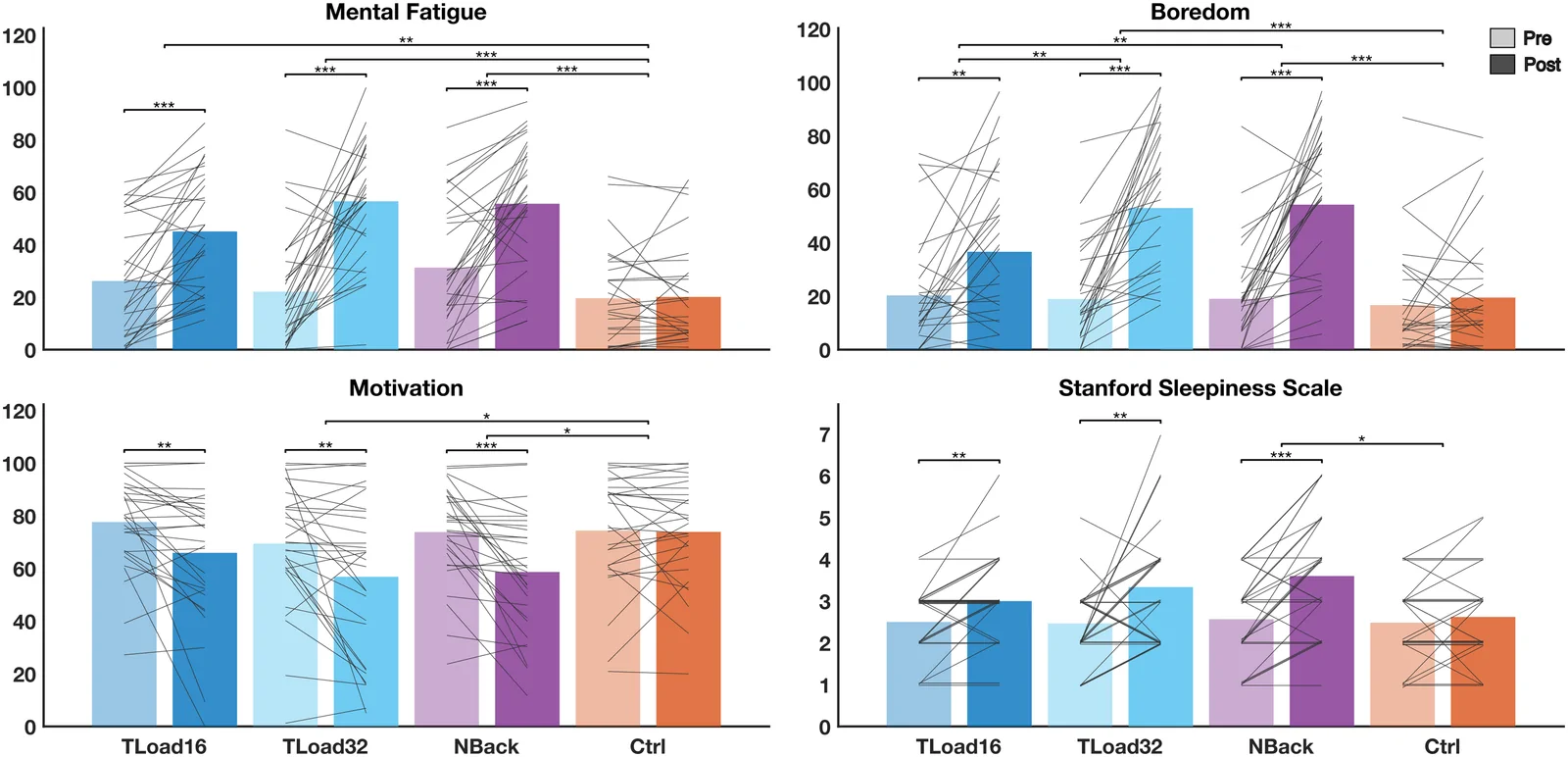
Results of the VAS subjective scales (mental fatigue, boredom, motivation, and sleepiness). Bars represent the mean across participants for pre (light) and post (dark). Each light grey line represents individual participants. *Note* in the sleepiness panel that the number of lines does not correspond to the number of participants and is instead the aggregate of the number of participants scoring a certain way. The pre-post significance levels and the significant differences between change scores are presented with horizontal black lines. * represents significance <0.01., ** represents significance <0.001., *** represents significance <0.0001

There was a significant effect of task ( $F\;\left(3,84\right)=16.05,p<0.0001,\eta^{2}=0.19$ ) on the reported levels of boredom, see Figure 2. Post hoc testing using the Bonferroni correction revealed significant differences between the change scores of the TLoad32 and N-Back tasks with the control task (M = 31.44 ( p=0.0002 ), 33.51 ( p< 0.0001 ), respectively), but not between the TLoad16 task and the control task (M = 13.96 ( p=0.18 )). The change scores of the TLoad16 task were significantly different from the change scores of TLoad32 and N-Back tasks (M = −17.49 ( p=0.0018 ), −19.55 ( p=0.0041 ), respectively).

The type of task had a significant effect on the levels of motivation ( ${Χ}^{2}\left(3\right)=10.78,p=0.01$ ), see Figure 2. Post hoc pairwise comparisons were conducted using the Durbin-Conover test with Holm corrections showed that the changes in motivation differed significantly between the TLoad32 and N-Back tasks and the control task ( p=0.034,p=0.05 , respectively), but not between any other tasks ( p>0.13) .

The type of task had a significant effect on the levels of sleepiness ( ${Χ}^{2}\left(3\right)=8.65,p=0.03$ ), see Figure 2. Pairwise comparisons showed that the changes in sleepiness were only significantly different between the N-Back and control tasks ( p<0.04) .

It was confirmed that none of the tasks had significantly different pre-scores across all subjective measures ( ${Χ}^{2}\left(3\right)=5.65,p=0.13$ ; ${Χ}^{2}\left(3\right)=3.22,p=0.36$ ; ${Χ}^{2}\left(3\right)=3.30,p=0.35$ ; ${Χ}^{2}\left(3\right)=1.51,p=0.68$ for mental fatigue, boredom, motivation, and sleepiness, respectively).

For RSME, Mauchly’s test of sphericity showed a significant difference in variation between the group differences: W(5)=0.55,p=0.007 ; therefore, the Huynh-Feldt correction was used. This test revealed a significant different score between different tasks ( $F\left(2.42,67.93\right)=93.43,p<0.0001,\eta^{2}=0.61$ ), see Figure 3. Post hoc testing using the Bonferroni correction revealed that RSME was significantly different between all experimental tasks and the control task (M = 6.22 ( p< 0.0001 ), M = 6.53 ( p < 0.0001 ), M = 6.08 ( p < 0.000 1) for TLoad16, TLoad32, and the N-Back tasks, respectively). There were no significant differences between the three mentally fatiguing tasks.

**Figure 3.**
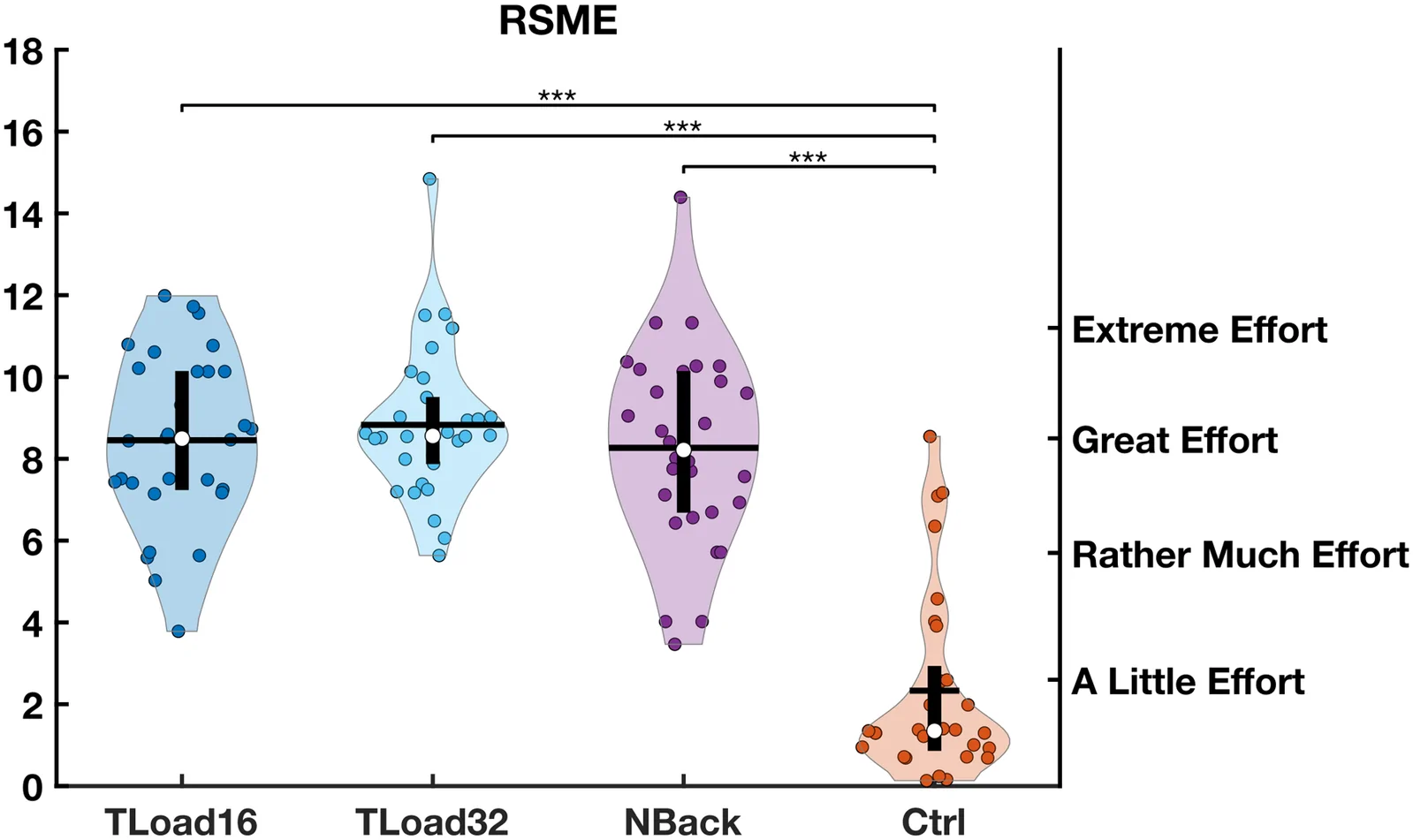
Rating Scale Mental Effort. The *y*-axis is here presented in cm. Each dot represents one participant. The black horizontal line represents the mean, the white dot, the median and the shape of the violin represents the spread of the data. *** represents significance <0.0001

In the NASA-TLX, to avoid any mathematical bias (Bolton et al., 2023), each domain was investigated separately, see Supplemental Material. For all domains, the type of task showed a significant effect. The control task was for all domains different from the mentally fatiguing tasks.

### Objective Measures

The change scores of the mean reaction time (mPVT) were significantly different across tasks ( $F\;\left(3,84\right)=6.65,p=0.0004,\eta^{2}=0.07$ ), see Figure 4. Post hoc tests using the Bonferroni correction revealed a significant difference between both the TLoad32 and N-Back tasks and the control task (M=19.53 (p=0.02),M=22.11 (p=0.010)) . No other significant differences were found. In contrast, the standard deviation of the reaction time was not different across tasks ( ${Χ}^{2}\left(3\right)=4.2,p=0.24$ ).

**Figure 4.**
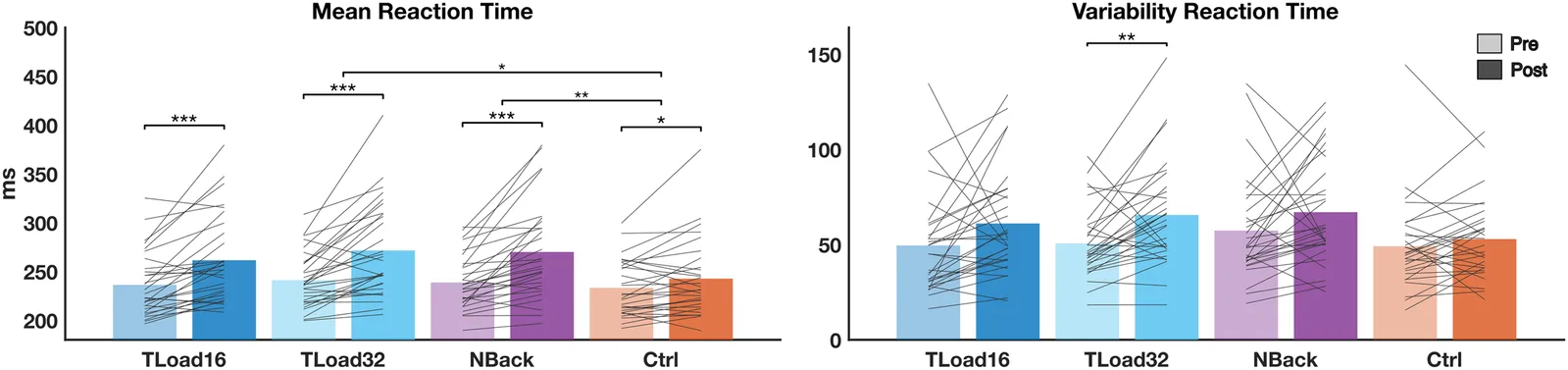
Reaction time. (A) Mean reaction time in ms. Bars represent the mean across participants for pre (light) and post (dark) and the light grey lines each participant. The significance levels represent the difference between pre-and post or between change scores of each task. * represents significance <0.01, ** represents significance <0.001, *** represents significance <0.0001

## Discussion

The aim of this study was to investigate which mentally fatiguing task could effectively induce mental fatigue, while simultaneously assessing boredom, loss of motivation, and sleepiness. To this end, a within-participant design was used involving 30 participants after one drop-out.

### Subjective Measures

The results of the VAS of mental fatigue showed that all three mentally fatiguing tasks induced mental fatigue (i.e., the average subjective feelings post-task were larger than the subjective feelings pre-task), and that the change scores for all of them were greater than the change scores of the control task. For all tasks, however, some participants improved their state of mental fatigue from before to after the task (i.e., the subjective feelings of mental fatigue reduced). This could originate from either an altered understanding of the actual levels of mental fatigue post-test, or an actual reduction in mental fatigue due to not being sufficiently motivated to fulfill the task. The results also show that the 32 minute-version of the TloadDback task was the one leading to the largest average increase in the subjective feelings of mental fatigue.

We found that on average all mentally fatiguing tasks led to an increase in sleepiness, an increase in boredom and a decrease in motivation, from pre-task to post-task. However, the change score (i.e., the difference pre-to-post) did not differ significantly between the TLoad16 task and the control task in the boredom and motivation measures, while it did for the other two mentally fatiguing tasks. For sleepiness, only the change score of the N-Back task resulted in increased sleepiness compared to the control task. This suggests that the longer the task, the more bored the participants were, despite the relatively high workload. Similar for motivation: motivation decreased with increased task duration, adversely affecting the participants’ willingness to continue with the task. Since the Tload16 task does not result in a significant increase of boredom, loss of motivation, and sleepiness compared to a control task, it is relevant to use if one seeks to objectively measure the effects of mental fatigue without any associated changes. Additionally, an individualized task has the advantage that the workload is similar across participants and the short duration of the task means that it is easy to administer during a workday or as one out of a battery of tasks. However, if one is more interested in maximizing the induced changes in the reaction time and maximise mental fatigue, then the TLoad32 and NBack task might be more suitable.

As for motivation, it should be pointed out that this is also affected by the reward structure and potential penalties of performance loss. For example, the neurobiological model of Boksem and Tops (2008) highlights the importance of brain structures involved in motivation, potentially mediated by fluctuations in dopamine levels. As such, our occupational examples cited in the Introduction (flight leaders and surgeons) are likely strongly motivated to perform their tasks to the best of their ability, thus being more resilient to the detrimental effects of prolonged cognitive activity. In our experiment we tried to keep motivation high by the prospect of a small monetary reward, but this is arguably far removed from daily challenges faced by certain occupations like the ones mentioned.

The two only post-task outcome measures, RSME and the NASA-TLX (see Supplemental Material) are measures of the mental workload/effort perceived by the participant after completion. For all measures, we only found differences between the control task and the mentally fatiguing tasks, which indicates that all three mentally fatiguing tasks were effective in inducing feelings of effort/mental fatigue. The lack of differences between the three mentally fatiguing tasks indicates that RSME and NASA-TLX are not sensitive enough to tease apart sleepiness and boredom from feelings of mental fatigue.

In this study we compared three mentally fatiguing tasks, the individualized TloadDback task of 16 and 32 minutes, respectively, with a N-back task of 32 minutes. The two tasks were chosen to balance complexity and time. Borragan et al. (2017) compared different versions of the TloadDback task (high and low cognitive load) and found that the high cognitive load version (as used here) was the most efficient task to balance sleepiness and mental fatigue. O'Keeffe et al. (2020) also compared different tasks in terms of duration of the task and found, like Borragan et al. (2017), that the shorter and more complex task led to more mental fatigue but less sleepiness. However, they failed to control for the fact that both task duration and complexity varied between tasks. In the present study we show that duration and complexity comes with a trade off in terms of a degree of sleepiness and boredom, and choosing a task and a duration might influence this trade off.

### Objective Measures

A 3-min version of the PVT task was used to assess reaction time pre- and post-task. The PVT was originally developed for 10-min use (Dinges & Powell, 1985). Later studies have shown that a shortened version of 3 minutes is reliable in sleep deprivation research (Basner et al., 2011); this shortened version has been used successfully in previous mental fatigue research (Rubio-Morales et al., 2022; Smith et al., 2019). Thus, to decrease the time spent on assessment, this shorter version was used. Using reaction time in combination with subjective measures strengthens the finding that mental fatigue was in fact induced.

The reaction time measures showed that all tasks resulted in an increase in mean reaction time from before the task to after the task. The reaction time increased by 25 ms (from 236 ms to 261 ms) for the 16-min version of the TloadDback task (TLoad16), whereas the increase was only 11 ms (from 233 ms to 242 ms) for the control task. While the increase may not appear large, an increase in reaction time of 25 ms is larger than the 7-10 ms increase Smith et al. (2019) found over three different 45-min tasks.

Only the 32-min version of the TloadDback task (Tload32) additionally resulted in an increase in the variability of reaction time. Reaction time variability is a measure for how consistently the task is performed, and can be a sign of longer lapses (<1 s). It is known that subjective increases in mental fatigue can preceed changes in reaction time (Smith et al., 2019; van der Linden, 2011), but since we found significant differences pre-and post task for all tasks, we do not expect this to be the case. This finding is relevant when choosing a task when doing further research into this area as for some an increase in variability of reaction time might be paramount.

## Control Task

The choice to include three documentaries in the control task was made to avoid that the documentary would lead to increased boredom or sleepiness for some participants, as was found in previous studies (O'Keeffe et al., 2020; Weissinger et al., 2024). Therefore, participants were encouraged to choose the documentary they would enjoy the most. That the documentary did not result in boredom suggests that a choice of three different documentaries is sufficient to avoid participants from becoming excessively bored or engaged. The control task was inherently different from the mentally fatiguing tasks as no engagement was needed. However, this approach has been widely used (Hachard et al., 2020; Martin et al., 2019; O'Keeffe et al., 2020; Smith et al., 2019; Weissinger et al., 2024). An alternative would have been a low-effort task like either of the mentally fatiguing tasks, but this could potentially have confounded the findings as it might not have been possible to say that the control task did not induce some form of mental fatigue. Utilizing a completely passive task (documentary viewing), but one that participants had some control over (in terms of choice), led the control task to serve as a proper control no matter the mentally fatiguing task chosen.

## Conclusion

The present study confirmed the findings of O'Keeffe et al. (2020) that a 16-min individualized task is successful in inducing subjective mental fatigue without resulting in increases in boredom, loss of motivation, or sleepiness, but still resulting in significant objective performance decrements in mean reaction time. This still holds when comparing an individualized 32 minute-version of the TloadDback Task to a task of similar duration but differing complexity. This finding is another step towards being able to reliably induce mental fatigue and assess its effects in behavioral experiments and a shorter or longer task can be chosen while balancing the effects also on boredom and sleepiness.

## Supplemental Material

Supplemental Material - A Comparison of Experimental Methods to Induce Mental Fatigue Supplemental Material for A Comparison of Experimental Methods to Induce Mental Fatigue by Margit M. Bach, Kerstin Brandstetter, John Stins, Peter J. Beek in Perceptual and Motor Skills.

## Data Availability

All data and the updated code of the task is available on: https://osf.io/cdpvx/.
