# Supplementary material for: A Comparison of Experimental Methods to Induce Mental Fatigue
Source: Percept Mot Skills. 2026 Feb 11;133(6):1763–82. doi: 10.1177/00315125261420694 (PMC13724811; doi:10.1177/00315125261420694)
Supplement: Supplementary file 1 [file sj-pdf-1-pms-10.1177_00315125261420694.pdf]

Supplemental Material

For Mental Demand, Mauchly's test of sphericity showed a significant difference in variation between the group differences: $W(5) = 0.59, p = 0.015$, therefore the Huynh-Feldt correction was used. This showed that there was a significant different score between different tasks ($F = (2.67,74.67) = 82.20, p < 0.0001, \omega^2 = 0.56$). Post hoc testing using the Bonferroni correction revealed that Mental Demand was significantly different between all experimental tasks and the control task (M = 53.38 ($p < 0.0001$), M = 55.62 ($p < 0.0001$), M = 47.14 ($p < 0.0001$) for TLoad16, TLoad32, and the N-Back tasks respectively). There were no significant differences between the three mentally fatiguing tasks.

The type of task had a significant effect on the levels of Physical Demand ($\mathrm{X}^2(3) = 34.76, p < 0.0001$). Pairwise comparisons using the Bonferroni correction revealed that Physical Demand was significantly different between all experimental tasks and the control task ($p < 0.0001$, $p < 0.0001, p = 0.04$ for TLoad16, TLoad32, and the N-Back tasks respectively). There were no significant differences between the three mentally fatiguing tasks.

The type of task had a significant effect on the levels of Temporal Demand ($\mathrm{X}^2(3) = 44.23, p < 0.0001$). Pairwise comparisons using the Bonferroni correction revealed that Temporal Demand was significantly different between all experimental tasks and the control task ($p < 0.0001$, $p < 0.0001$, $p = 0.0002$ for TLoad16, TLoad32, and the N-Back tasks respectively). There were no significant differences between the three mentally fatiguing tasks.

There was a significant effect of task ($F = (3, 84) = 23.10, p < 0.0001, \omega^2 = 0.34$) on the reported levels of Performance. Post hoc testing using the Bonferroni correction revealed that Performance was significantly different between all experimental tasks and the control task (M = 38.00 ($p < 0.0001$), M = 38.21 ($p < 0.0001$), M = 39.14 ($p < 0.0001$) for TLoad16, TLoad32, and the N-Back tasks respectively). There were no significant differences between the three mentally fatiguing tasks.

The type of task had a significant effect on the levels of Effort ($\mathrm{X}^2(3) = 54.69, p < 0.0001$). Pairwise comparisons using the Bonferroni correction revealed that Effort was significantly different between all experimental tasks and the control task ($p < 0.0001, p < 0.0001, p = 0.0003$ for TLoad16, TLoad32, and the N-Back tasks respectively). There were no significant differences between the three mentally fatiguing tasks.

The type of task had a significant effect on the levels of Frustration ($\mathrm{X}^2(3) = 50.46, p < 0.0001$). Pairwise comparisons using the Bonferroni correction revealed that Frustration was significantly different between all experimental tasks and the control task ($p < 0.0001, p <$

0.0001, $p <$ 0.0001 for TLoad16, TLoad32, and the N-Back tasks respectively). There were no significant differences between the three mentally fatiguing tasks.

Values are shown in Supplementary Figure 1.

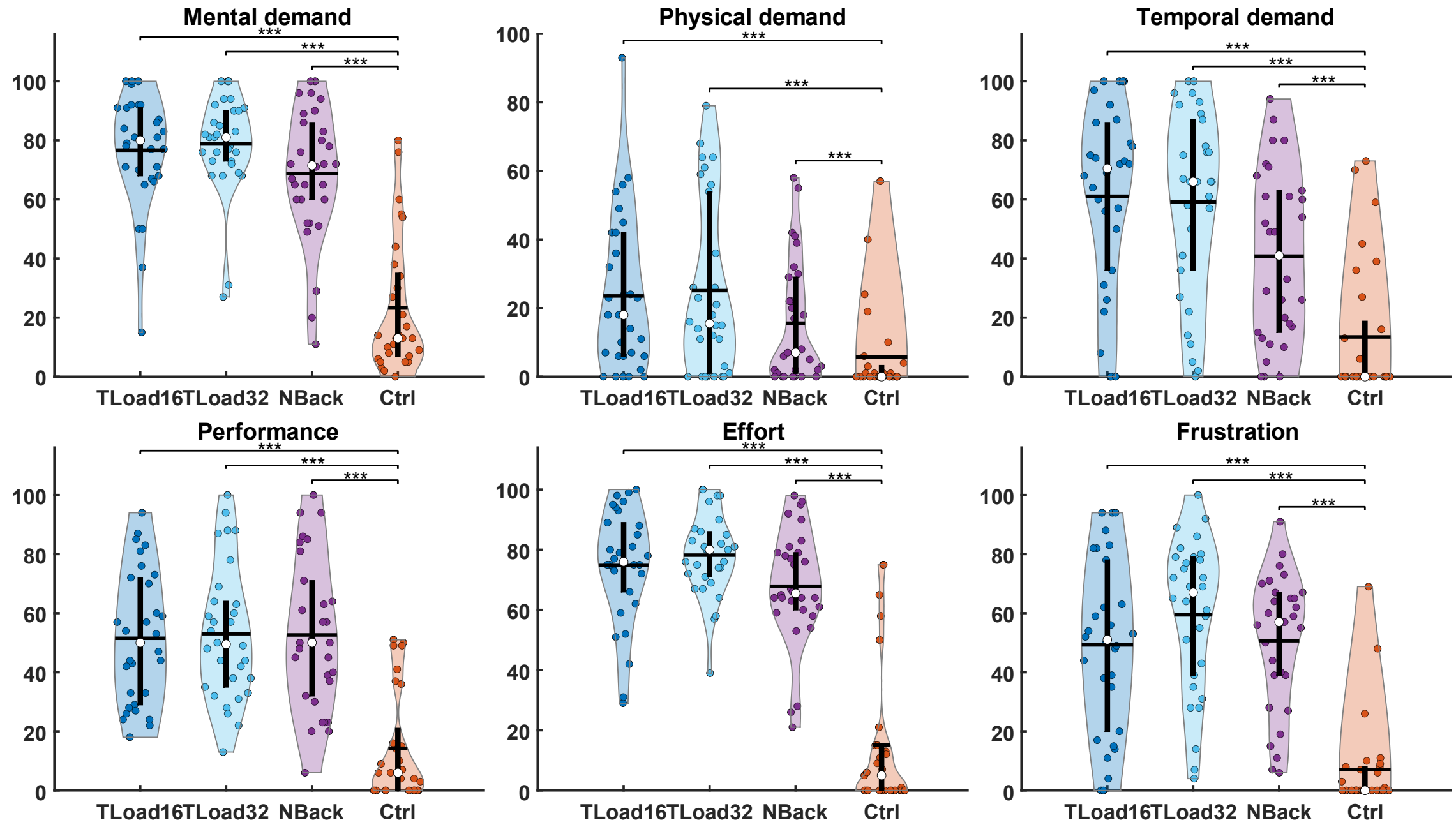

*Figure S1: NASA-TLX. Each dot represents one participant. The black horizontal line represents the mean, the white dot, the median and the shape of the violin represents the spread of the data. * represents significance < 0.01, ** represents significance < 0.001, *** represents significance < 0.0001.*
